# Characterization of the skin microbiome in normal and cutaneous squamous cell carcinoma affected cats and dogs

**DOI:** 10.1128/msphere.00555-23

**Published:** 2024-03-26

**Authors:** Jacoba I. Bromfield, Julian Zaugg, Rodney C. Straw, Julia Cathie, Annika Krueger, Debottam Sinha, Janin Chandra, Philip Hugenholtz, Ian H. Frazer

**Affiliations:** 1 Frazer Institute, Faculty of Medicine, The University of Queensland, Woolloongabba, Queensland, Australia; 2 Australian Centre for Ecogenomics, University of Queensland, St Lucia, Queensland, Australia; 3 Brisbane Veterinary Specialist Centre and the Australian Animal Cancer Foundation, Albany Creek, Queensland, Australia; Nanjing University of Chinese Medicine, Nanjing, Jiangsu, China

**Keywords:** skin microbiome, animal models, veterinary microbiology, skin cancer, squamous cell carcinoma, *Staphylococcus*, SSU rRNA, actinic keratosis

## Abstract

Human cutaneous squamous cell carcinomas (SCCs) and actinic keratoses (AK) display microbial dysbiosis with an enrichment of staphylococcal species, which have been implicated in AK and SCC progression. SCCs are common in both felines and canines and are often diagnosed at late stages leading to high disease morbidity and mortality rates. Although recent studies support the involvement of the skin microbiome in AK and SCC progression in humans, there is no knowledge of this in companion animals. Here, we provide microbiome data for SCC in cats and dogs using culture-independent molecular profiling and show a significant decrease in microbial alpha diversity on SCC lesions compared to normal skin (*P ≤* 0.05). Similar to human skin cancer, SCC samples had an elevated abundance of staphylococci relative to normal skin—50% (6/12) had >50% staphylococci, as did 16% (4/25) of perilesional samples. Analysis of *Staphylococcus* at the species level revealed an enrichment of the pathogenic species *Staphylococcus felis* in cat SCC samples, a higher prevalence of *Staphylococcus pseudintermedius* in dogs, and a higher abundance of *Staphylococcus aureus* compared to normal skin in both companion animals. Additionally, a comparison of previously published human SCC and perilesional samples against the present pet samples revealed that *Staphylococcus* was the most prevalent genera across human and companion animals for both sample types. Similarities between the microbial profile of human and cat/dog SCC lesions should facilitate future skin cancer research.

**IMPORTANCE:**

The progression of precancerous actinic keratosis lesions (AK) to cutaneous squamous cell carcinoma (SCC) is poorly understood in humans and companion animals, despite causing a significant burden of disease. Recent studies have revealed that the microbiota may play a significant role in disease progression. *Staphylococcus aureus* has been found in high abundance on AK and SCC lesions, where it secretes DNA-damaging toxins, which could potentiate tumorigenesis. Currently, a suitable animal model to investigate this relationship is lacking. Thus, we examined the microbiome of cutaneous SCC in pets, revealing similarities to humans, with increased staphylococci and reduced commensals on SCC lesions and peri-lesional skin compared to normal skin. Two genera that were in abundance in SCC samples have also been found in human oral SCC lesions. These findings suggest the potential suitability of pets as a model for studying microbiome-related skin cancer progression.

## INTRODUCTION

Squamous cell carcinomas (SCCs) are common malignant cancers in older cats, accounting for 15% of feline skin tumors and 70% of feline oral tumors (1). Presentation of cutaneous SCC typically occurs in sparsely haired areas, such as the ear pinnae, nasal planum, eyelids, and on predominantly white short-haired cats, due to the lack of pigmentation and exposure to chronic UV irradiation (1, 2). Cutaneous SCC is also the most common skin cancer in dogs, presenting in similar sites to cats but extending to the abdominal and perianal regions. However, dogs typically have a lower metastatic rate compared to cats, and this type of cancer is relatively rare compared to other types of canine cancers (3, 4). Progression of these tumors in both cats and dogs are often insidious as they appear as non-healing scabs that ulcerate with subsequent swelling, erythema, tissue erosion, and are typically not diagnosed until the tumor is late stage (1, 5).

The relationship between skin disease and the skin microbiome has gained increasing interest in the last decade, with recognition that a healthy skin microbiome is crucial for maintaining skin homeostasis (6). A healthy skin microbiome protects against invading pathogens, contributes to immune system education, and enables the breakdown of natural products (7). The skin microbiome has a complex relationship with the immune system, as a healthy cutaneous microbiome can maintain immune homeostasis of the animal, whereas microbial dysbiosis can lead to opportunistic pathogens colonizing the perturbed area (8).

Microbial dysbiosis is observed on cutaneous SCC lesions in humans. Several studies have suggested a link between skin microbiota and cutaneous SCC progression, whereby *Staphylococcus aureus* has been found in high relative and absolute abundance on actinic keratosis lesions, and even more so on cutaneous SCCs (9–12). The presence of pathogenic bacteria on the skin can cause an inflammatory response, and inflammation is widely recognized as a factor in promoting tumorigenesis (13, 14). In the study of Krueger et al., *S. aureus* isolated from human SCC lesions were reported to contribute to DNA damage in healthy human keratinocytes cultured *in vivo* from abdominal and foreskin tissues (15). Exposure to the secretome from AK and SCC *S. aureus* isolates upregulated several SCC biomarkers, which subsequently induced oxidative stress and downregulated DNA repair, leading to DNA damage and suggesting a potential mechanism contributing to SCC formation.

A translatable, replicable model for human skin microbiome studies with respect to cutaneous *S. aureus* colonization has not been established. As *S. aureus* is not a normal colonizer of mouse skin, it is challenging and highly labor intensive to maintain a colony on their skin (16, 17). In addition, previous studies that have successfully established an infection and developed a vaccine for *S. aureus* in mouse skin have failed in a clinical setting (18). However, staphylococcal species, including *S. aureus*, have been found as transient colonizers on healthy and diseased dogs and cats (19, 20).

Here, we provide microbiome data for cutaneous SCC compared to normal skin in cats and dogs using culture-independent molecular profiling to ascertain whether companion animals may be a good model for human AK to SCC progression investigating bacterial pathogenicity.

## MATERIALS AND METHODS

### Ethics and animal cohort

**Fig 1:**
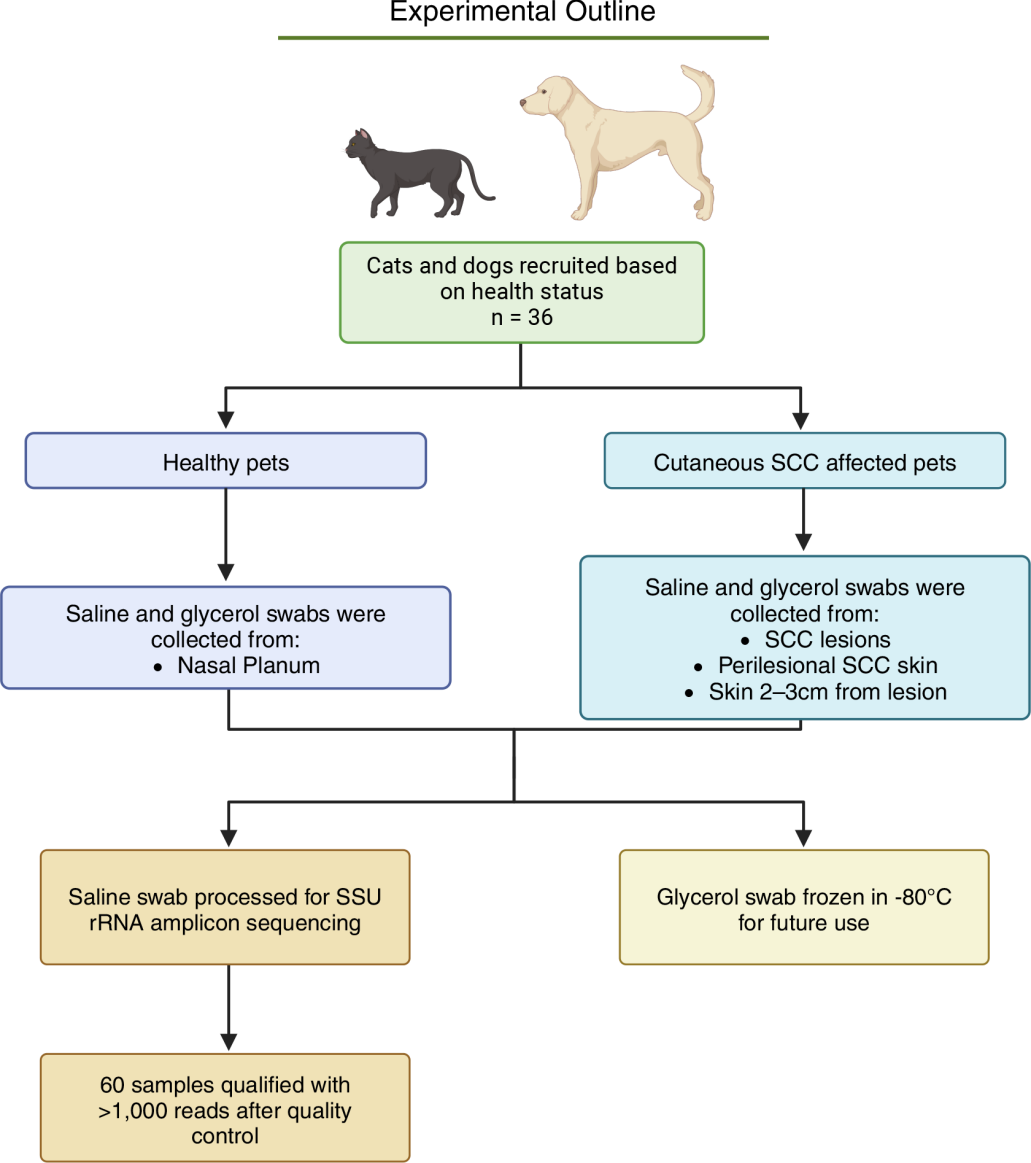
Outline of experimental steps for this study.

All experimental procedures were approved by the Animal Ethics Committee of the University of Queensland prior to commencement of the study (number: UQDI/188/19). A cohort of 36 domestic cats and dogs, encompassing various diets, ages, outdoor/indoor lifestyles, and both spayed/neutered and intact animals, were recruited (Fig. 1; Table S1). Pets that were recorded as indoor occupying were given supervised outdoor access each day; therefore, microbiome differences between indoor and outdoor lifestyles were not assessed due to these confounding factors and small sample size. The first animal was swabbed on 5 January 2020, and the final animal was swabbed on 20 June 2022. SCC lesions were present in 13 of the animals, while 23 had normal skin (no pre-/cancerous lesions observed). Lesions were identified by a veterinarian from specimen resections and an accompanying pathology report from Queensland Medical Laboratory (QML) Pathology. Predominantly, lesions were found on the nasal planum, with fewer located on the tail, nose, leg, neck, and abdomen (Fig. S1a and b).

### Sample collection, DNA extraction, and PCR

Three no-medium swabs (Sarstedt, Nümbrecht 51588, Germany) were taken from predominately elderly pets (age 6–17 years old, median 12 years) identified as having SCC lesions (Table S1). The first swab was from the identified SCC lesion, and the second and third swabs were adjacent to and 2–3 cm away from the lesion, i.e., perilesional (SCC_PL), respectively, in a sparsely haired location. The second and third swabs that were adjacent to and 2–3 cm away from the lesion were combined for analytical purposes and labeled as SCC_PL. Normal skin (NS) control swabs were taken from the nasal planum of the non-diseased cats and dogs in a cross-sectional manner, as there is less hair relative to other sections of the face (Fig. S2a and b). Each swab was replicated twice; one swab was placed in a saline solution and stored at −80°C until small subunit ribosomal RNA (SSU rRNA) sequencing, and the second swab was stored in a 50% glycerol solution for future analysis (Fig. 1).

Upon sample collection, the DNA from the swab samples was extracted using the DNeasy PowerSoil Pro Kit (Qiagen). SSU rRNA genes in the V6–V8 region were PCR amplified with universally conserved primers 926F (5′-AAACTYAAAKGAATTGRCGG-3′) and 1392R (5′-ACGGGCGGTGWGTRC-3′) at the Australian Centre for Ecogenomics, The University of Queensland.

### Amplicon data analysis and statistics

The primer sequence was removed from forward de-multiplexed reads using cutadapt (ver. 2.6) (21), with reads not containing the primer discarded (--discard-untrimmed). Using QIIME2 (ver. 2020.11.1) (22), reads were filtered, dereplicated, and chimeras identified and removed by DADA2 (--p-trunc-len 270) (23). Taxonomy was assigned to the resulting amplicon sequence variants (ASV) (24) by aligning each (classify-consensus-blast) against the non-redundant SILVA database (release 138) (25). All analyses were performed in R (ver. 4.3.0). ASVs that were not bacterial, fungal, or archaeal in origin, classified at below the phylum level, or that were classified as chloroplast or mitochondria, were discarded.

Skin has extremely low microbial biomass due to its dry and nutrient-poor conditions (26); it has ~1/100th the biomass of stool samples (27). Low microbial biomass makes obtaining high read depth from amplicon sequencing challenging. To retain as many samples as possible for analysis, we set a lower threshold of 1,000 reads, allowing 60 samples to be used for analysis (23 NS, 25 SCC_PL, and 12 SCC). These samples had read depths ranging from 1,411 to 72,842 reads (median 10,487).

To account for the compositional nature of the data, ASV counts were collapsed to the genus level and were robust centered log-ratio (rclr) (28) transformed with the decostand function in vegan (ver. 2.6–4) (29) prior to principal component analysis (PCA). PCA was performed using the rda function on rclr-transformed counts with Euclidean distances. Beta-diversity Jaccard dissimilarity values (presence/absence) were also calculated using vegan. To test for significant differences in microbial composition between groups, permutational multivariate analysis of variance (PERMANOVA) (30) was performed with the adonis2 function in vegan (permutations = 9,999). Permutation tests for homogeneity of multivariate dispersions (PERMDISP) were used to measure the dissimilarity in the variation of microbial communities (31) and performed using the betadisper function to infer if significant differences identified by PERMANOVA were potentially due to homogeneity differences between groups. Differentially abundant lineages were identified using a combination of ALDEx2 (ver. 1.28.1) (32) and LinDA (ver. 0.1.0) (33), and by comparing relative abundance distributions with Kruskal–Wallis tests followed by Benjamini and Hochberg (34)-corrected Dunn’s multiple comparison tests. Alpha diversity metrics Shannon (diversity) and Simpson (evenness), and number of observed taxa, were calculated using phyloseq (ver. 1.40.0) (35) at the genus level on samples rarefied to 3,000 reads as a trade-off between sample inclusivity and statistical power (leaving 23 NS, 20 SCC_PL, and 11 SCC samples for analysis). Significant differences in alpha diversity distributions were also determined through Kruskal–Wallis tests followed by Benjamini and Hochberg (34)-corrected Dunn’s multiple comparison tests. The core microbiome was calculated across all sample types and defined as those genera that appeared across all sample types and per type, were present in at least two samples, and had a relative abundance of 1% in at least one sample. The (core) microbiomes of the pet SCC and SCC_PL samples were also compared to equivalent human skin swab samples from immunocompetent subjects (11, 12). The core microbiome for the human samples was derived using the same methodology as the pet samples. Details on the processing of the human samples is provided in the supplementary material (Text S1). Core microbiome results were visualized as Venn diagrams created with the VennDiagram package (ver. 1.7.3). Ordination figures were created with ggplot2 (ver. 3.4.2) (36), ggnewscale (ver. 0.4.9), and vegan functions. Boxplots, stacked bar charts, and scatter plots were created with ggplot2 and ggnewscale. Heatmaps were created using ComplexHeatmap (ver. 2.16.0) (37).

## RESULTS

### Cat and dog SCC lesions have reduced diversity compared to normal skin

**Fig 2:**
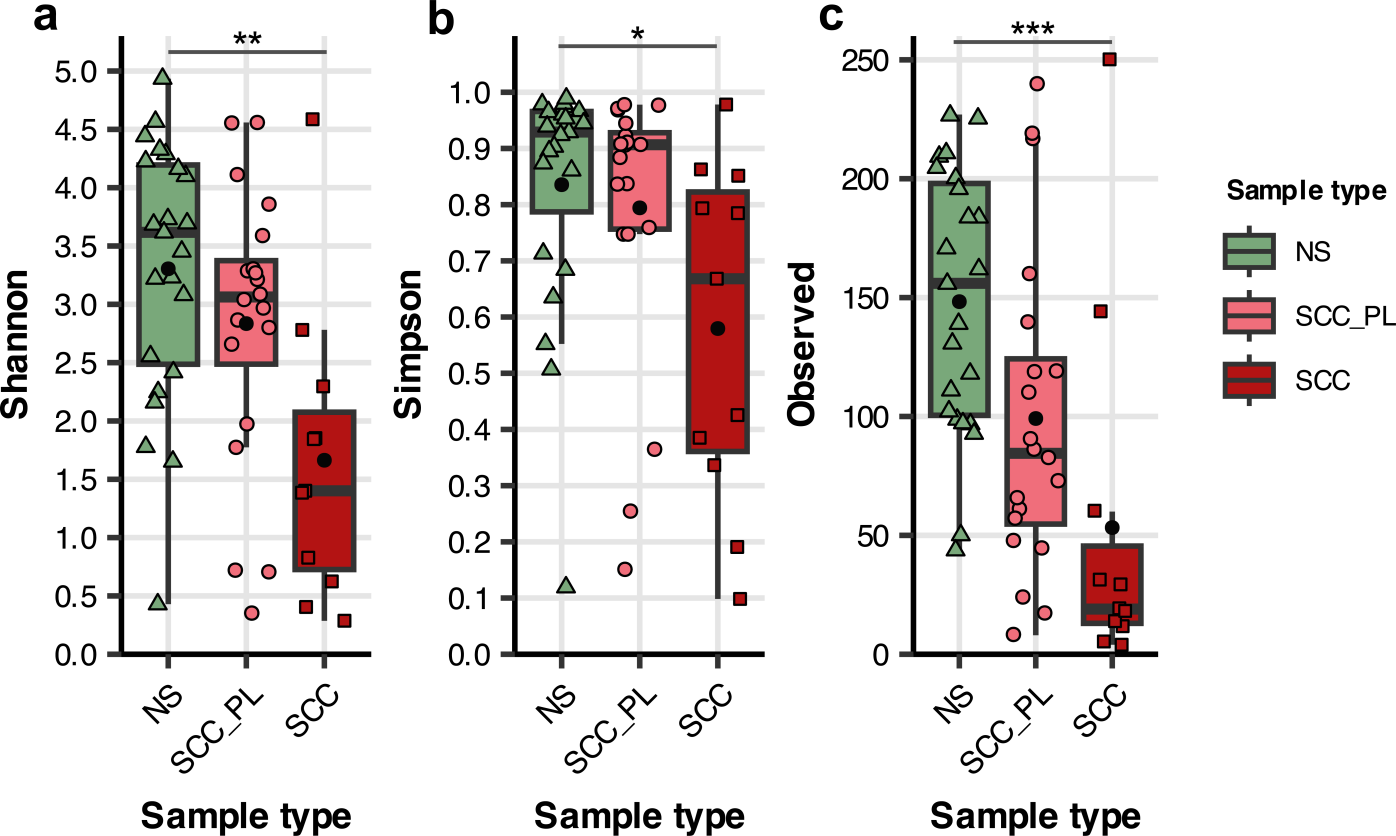
Alpha diversity in normal skin (NS), squamous cell carcinoma (SCC), and matching perilesional control (SCC_PL) swabs from the pet cohort. Tukey style box plots showing genus diversity across sample types, with diversity represented by three separate measures: Shannon (diversity), Simpson (evenness), and number of observed taxa. Bars indicate median ±1.5 × interquartile range, and the mean diversity for each sample type is indicated by a black dot. Significant differences between sample types are indicated by *P ≤ 0.05, **P ≤ 0.01, and ***P ≤ 0.001 as calculated via Dunn’s multiple comparison test.

Squamous cell carcinoma (SCC) swab samples had significantly lower bacterial diversity and fewer observed genera compared to normal skin (NS) samples (Fig. 2; Fig. S3 and S4) (*P ≤* 0.001–0.05). SCC samples had a mean Shannon value of 1.6, significantly lower than NS samples (mean = 3.3; *P ≤* 0.01), but not significantly lower than perilesional SCC (SCC_PL) samples (mean = 2.8) (Fig. 2A). NS and SCC_PL samples demonstrated high evenness, with a mean Simpson’s Index value of 0.84 and 0.8, respectively (Fig. 2B). By contrast, SCC samples had significantly lower Simpson values with a mean value of 0.58 (*P ≤* 0.05 vs NS), suggesting a less diverse microbial community dominated by fewer genera. This is supported by the significantly lower number of observed genera in SCC compared to NS samples (mean = 52.6 vs 147.8; *P ≤* 0.001) (Fig. 2C). No significant differences in diversity were observed between NS and SCC_PL samples, nor between SCC_PL and SCC samples. Furthermore, no significant differences were observed between samples adjacent to and 2–3 cm from lesions, suggesting that the reduction of bacterial diversity is restricted to the macroscopically abnormal skin (Fig. S4).

### Variation in microbial composition is primarily driven by individual, sample location, and sample type differences

**Fig 3:**
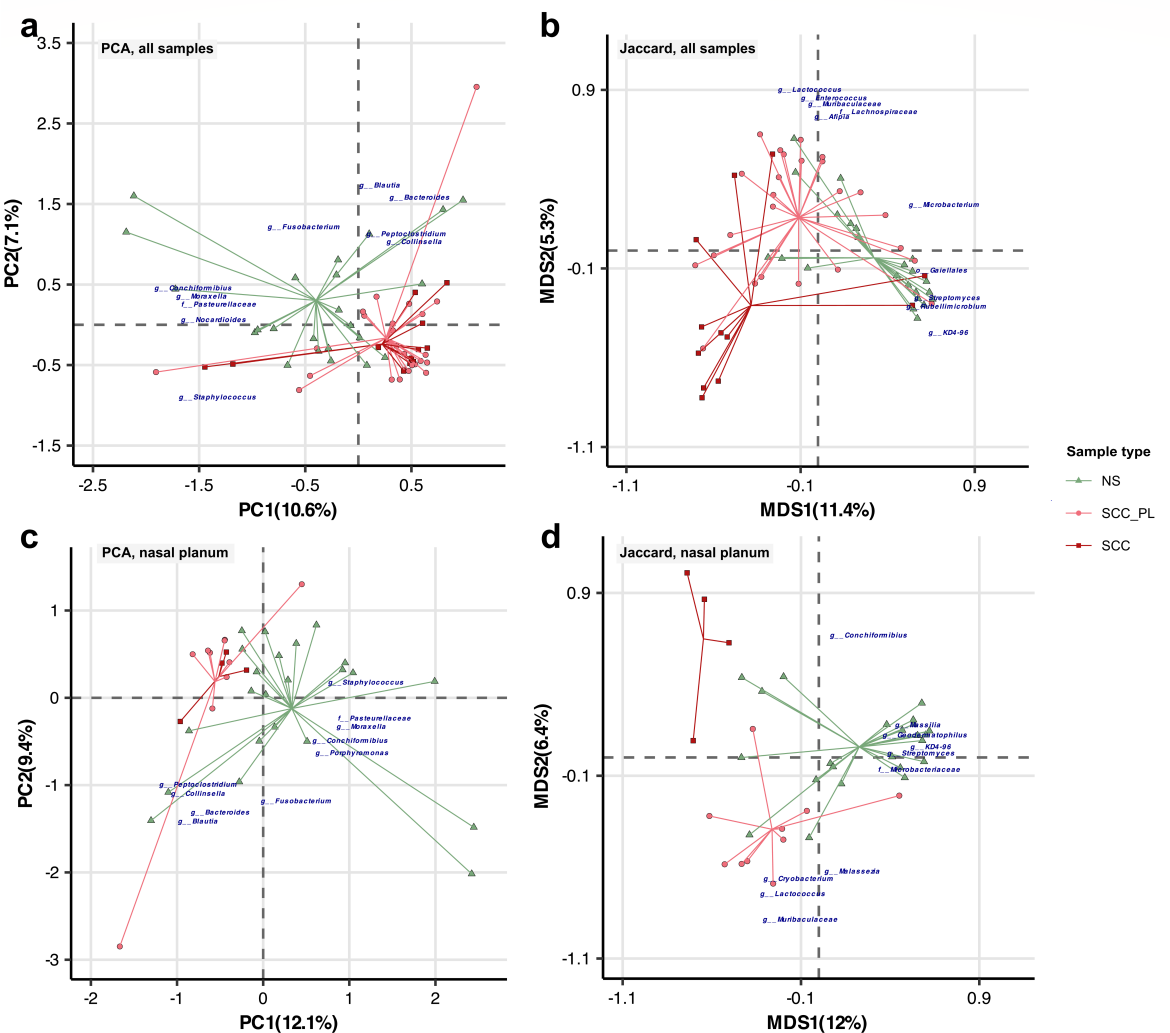
PCA of the Euclidean distances for robust centered log-ratio (rclr) values and ordination analysis of Jaccard dissimilarities, visualizing the variation in microbial community composition across skin swab samples for the pet cohort. Samples are annotated by sample type: normal skin (NS), squamous cell carcinoma (SCC), and matching perilesional controls (SCC_PL). (a, b) PCA and Jaccard ordinations based on all samples and (c, d) ordinations based on samples taken from the nasal planum.

PCA comparison of the Euclidean distances for robust centered log-ratio (rclr) values and for ordination analysis of Jaccard dissimilarities, each showed NS and SCC_PL/SCC samples forming separate clusters predominantly along the first ordination axis, albeit with notable variance among the samples (Fig. 3a and b). The five genera contributing most to the observed PC1 axis variance were *Conchiformibius* (7.0%), *Moraxella* (6.3%), *Nocardioides* (5.4%), *Staphylococcus* (5.1%), and an unassigned genus from the family *Pasteurellaceae* (5.0%). The highest contributions to the PC2 axis were from *Blautia* (10.5%), *Bacteroides* (8.7%), *Fusobacterium* (5.1%), *Peptoclostridium* (4.4%), and *Collinsella* (4.3%) (Table S2). PERMANOVA univariate analysis revealed that the individual pet contributed much to the variation (rclr: 72.1%, Jaccard: 61.8%), with smaller contributions from sample location (rclr: 16.6%, Jaccard: 18.5%) and sample type (rclr: 6%, Jaccard: 8.2%) (*P ≤* 0.001–0.05) (Table S3; Fig. S5a and e). This finding is consistent with previous veterinary and human studies, where a substantial portion of the microbial community variation was also attributed to inter-individual differences (12, 38). Pairwise PERMANOVA comparison of the sample types showed that the microbial composition of the SCC_PL and SCC samples was significantly different from NS (*P ≤* 0.001–0.01) (Table S3). The microbial communities for the sample types had dissimilar dispersions according to PERMDISP (*P ≤* 0.01) suggesting differences may partly be due to dispersion effects (dissimilarity between the variability in the microbial communities). Animal type, neuter status, and sex also contributed significantly to variation according to PERMANOVA (*P ≤* 0.001–0.05), though these variables contributed minimally to the overall variation (rclr: 2.9%, 2.6%, and 2.5%, respectively; Jaccard: 3.7%, 2.1%, and 2.1%) (Fig. S5b through d and f through h; Table S3).

Similar to the results from the inclusion of all sample locations, PCA and Jaccard dissimilarities of nasal planum swabs showed broad separation of NS and SCC_PL/SCC sample types (Fig. 3c and d). Again, PERMANOVA analysis indicated that the individual pet accounted for most of the variation (rclr: 83.0%, *P ≤* 0.01; Jaccard: 72.3%), with lower contributions from sample type (rclr: 7.3% *P ≤* 0.05, Jaccard: 12.4%; *P ≤* 0.001), neuter status (rclr: 3.9%, Jaccard: 3.3%), animal type (rclr: 3.4%, Jaccard: 3.8%; *P ≤* 0.05), and sex (rclr: 3.2%, Jaccard: 2.8%) (Fig. S6a through f, Table S3). A pairwise comparison indicated that NS and SCC_PL were significantly different (*P ≤* 0.05). The corresponding PERMDISP analysis was significant for sample type (*P ≤* 0.01), again suggesting that differences may be due in part to dispersion effects.

In summary, and consistent with findings from human cohorts, most variation in the skin microbiome is due to inter-individual differences; however, there are small but significant differences between sample types, i.e., between SCCs and normal skin.

### Differential abundance and distribution of microbial lineages between sample types

**Fig 4:**
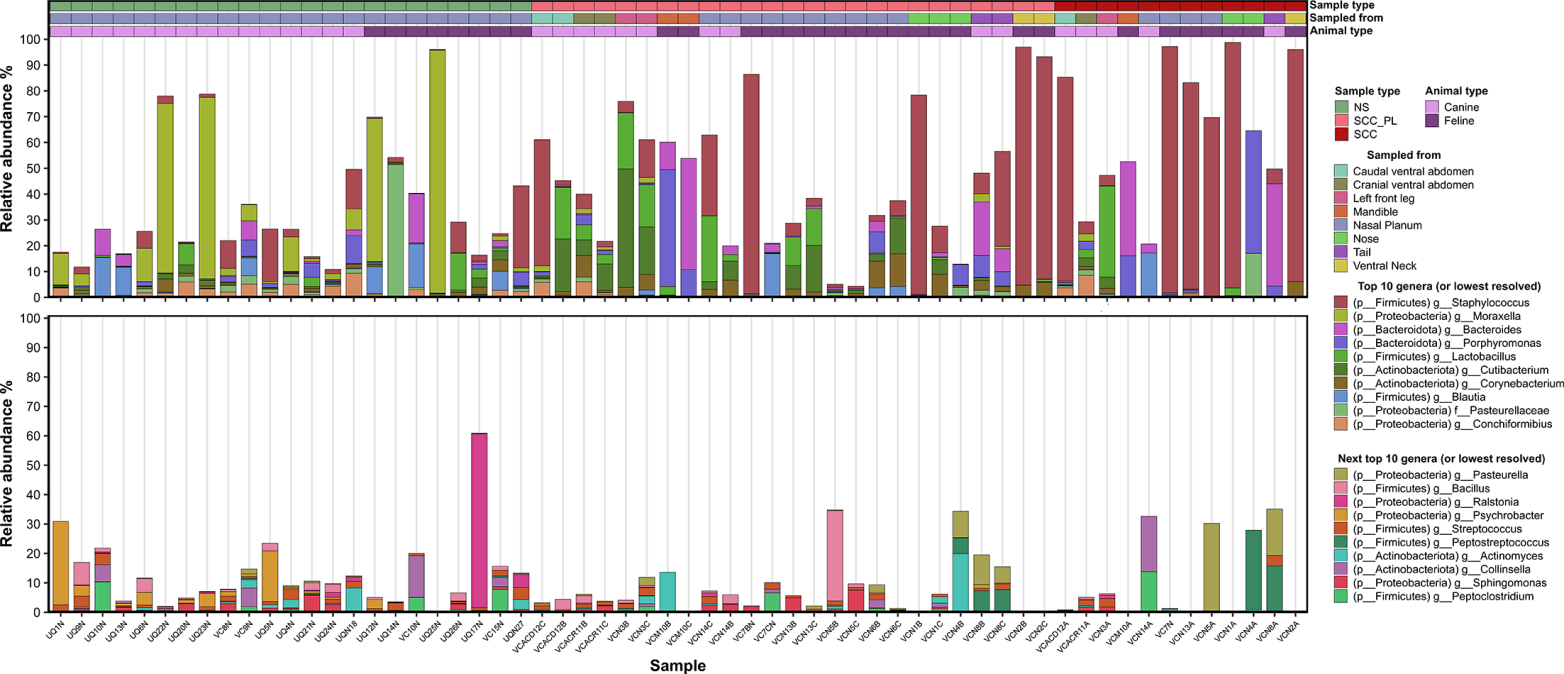
Relative microbial abundances in NS, SCC_PL, and SCC swab samples from individual pets. Relative abundances of the 20 most abundant skin microbes by mean abundance across swabs from normal skin (NS), squamous cell carcinoma (SCC), and matching perilesional controls (SCC_PL) from individual pets. Values are calculated from normalized SSU rRNA read counts collapsed to the genus level.

**Fig 5:**
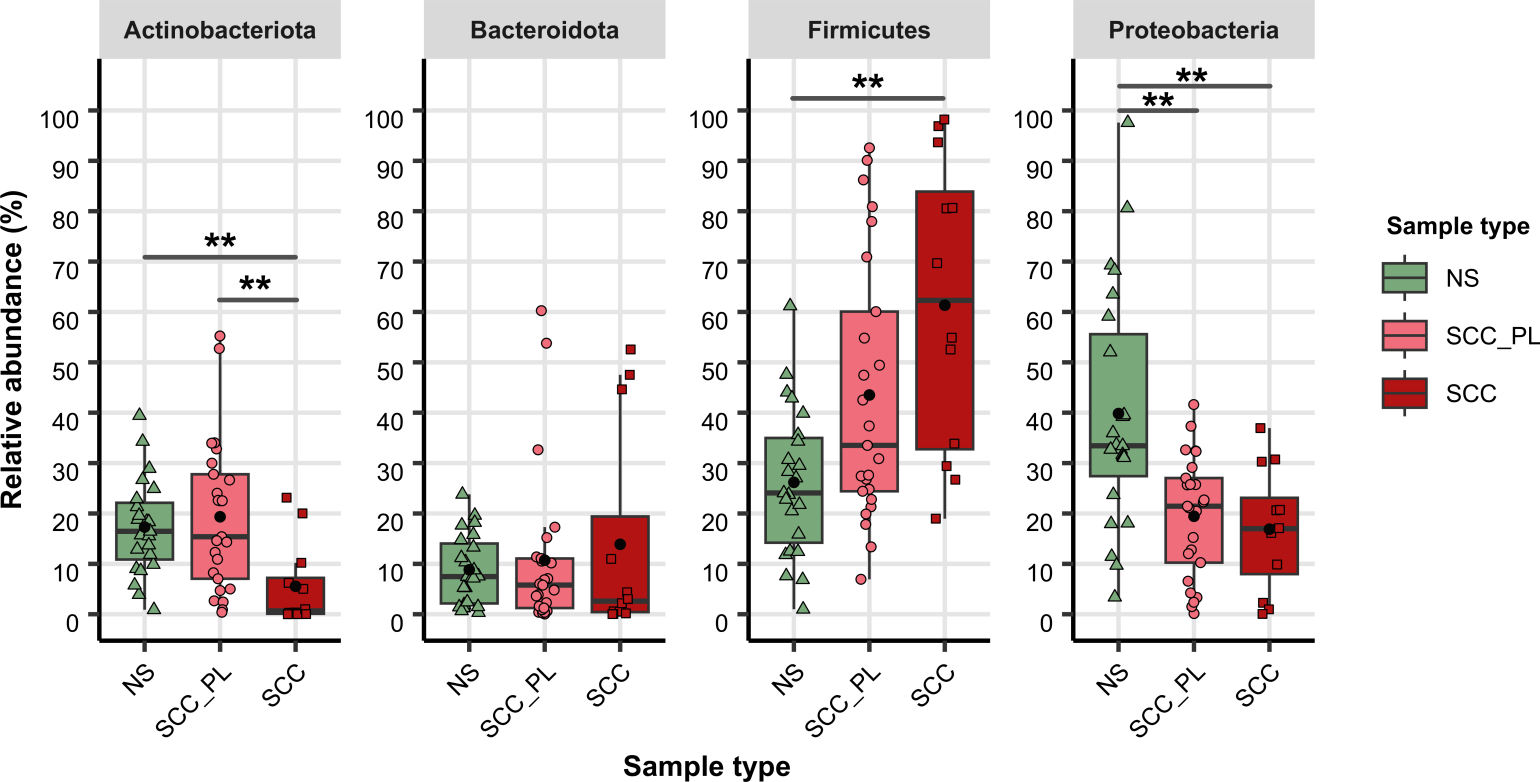
Relative abundance of high-abundance phyla across sample types. Tukey-style box plots showing the relative abundances (normalized SSU rRNA read counts) of phyla with a mean relative abundance ≥5% in at least one sample type, from swab samples from individual pets. Bars indicate the median ±1.5 × interquartile range, and the mean relative abundance for each sample type is indicated by a black dot. Significant differences between sample types, calculated by Dunn’s multiple comparison tests, are indicated by *P ≤ 0.05, **P ≤ 0.01, and ***P ≤ 0.001.

**Fig 6:**
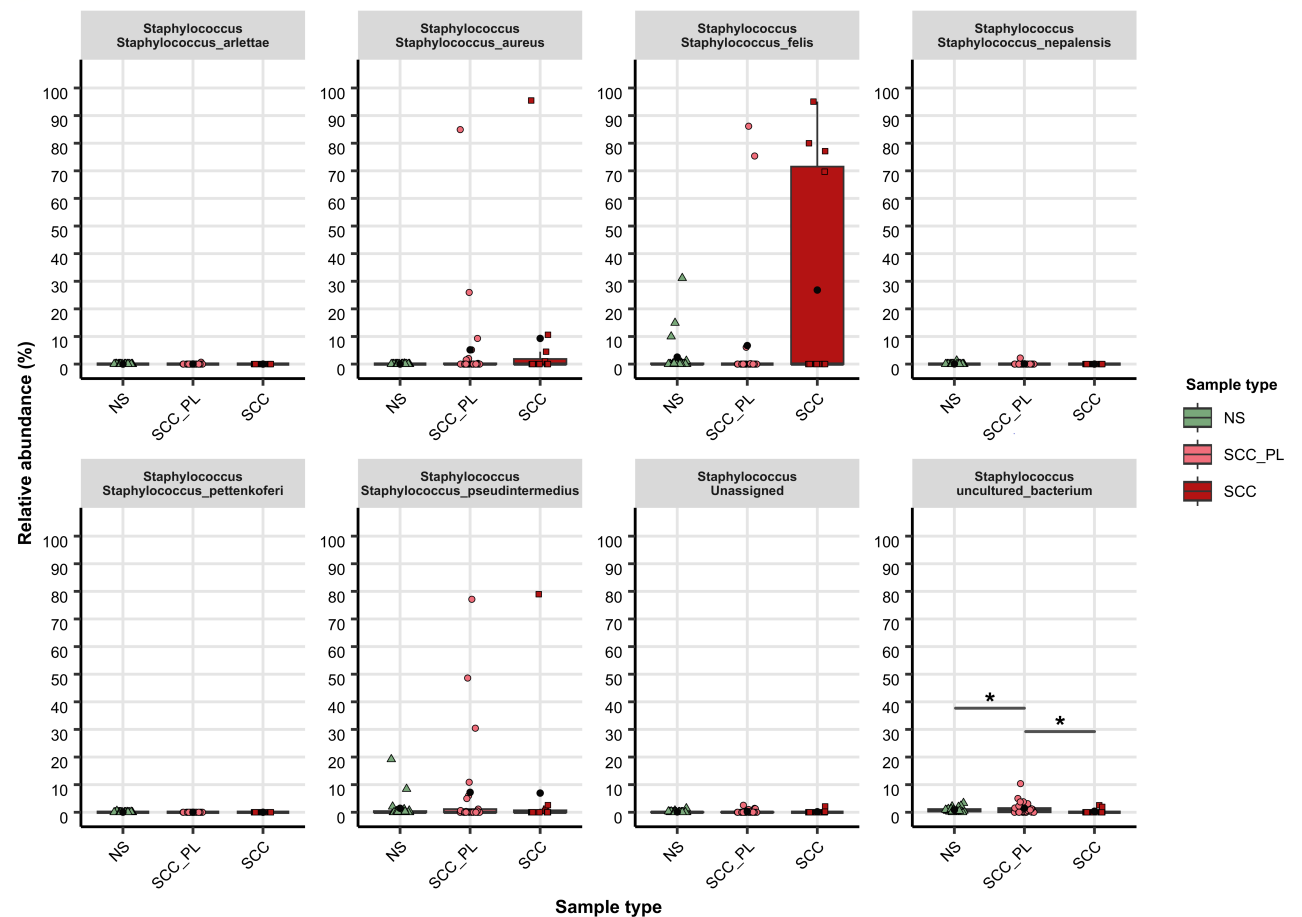
Relative abundance of staphylococcal species across sample types. Tukey-style box plots showing the relative abundances (normalized SSU rRNA read counts) of the staphylococcal species in swab samples from individual pets across sample types. Bars indicate the median ±1.5 × interquartile range, and the mean relative abundance for each sample type is indicated by a black dot.

**Fig 7:**
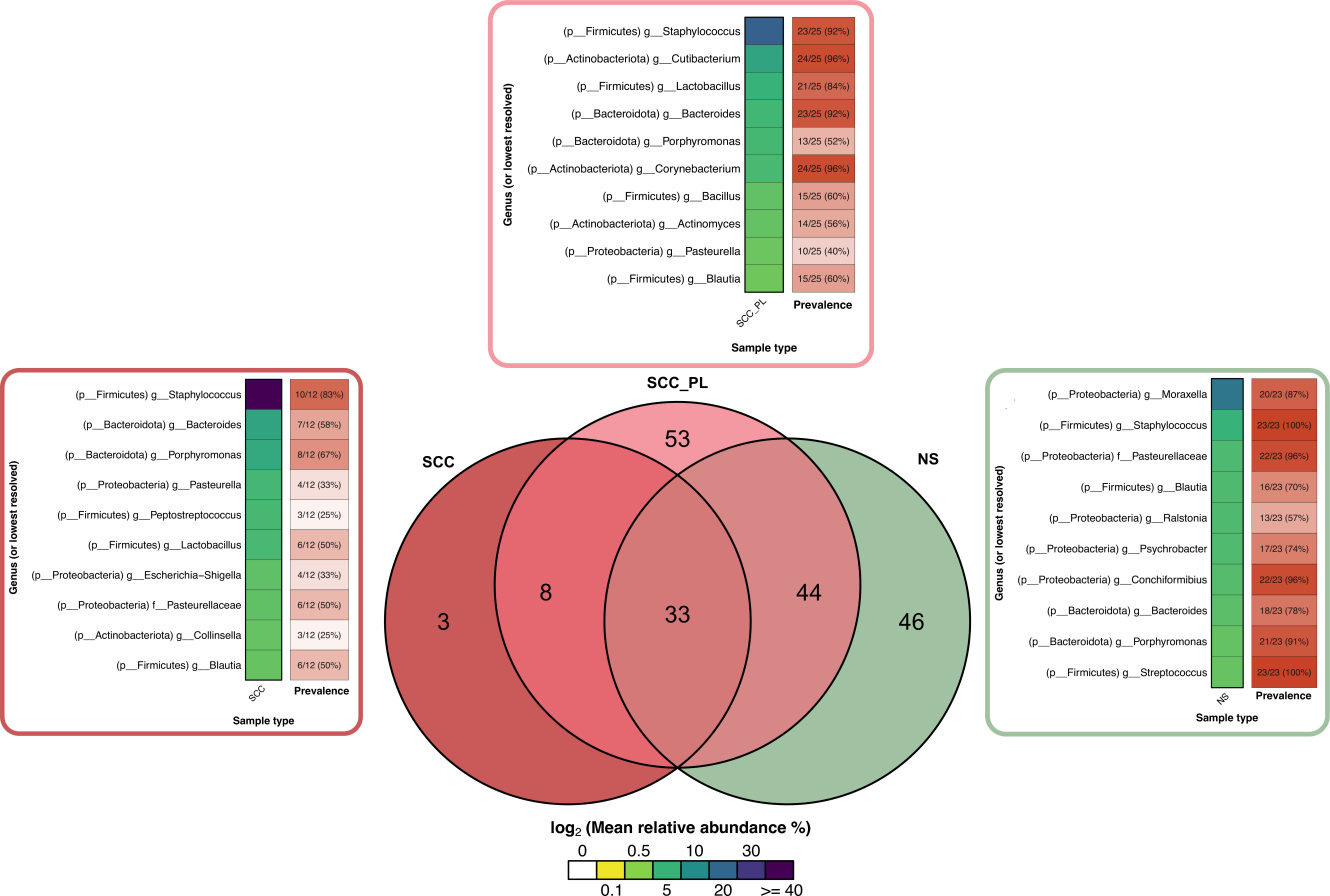
Venn diagram showing the number of shared microbes across SCC, SCC_PL, and NS sample types. The core microbiome was calculated across all sample types and defined as those genera that appeared across all sample types and per type, were present in at least two samples, and had a relative abundance of 1% in at least one sample. The mean relative abundances of the top 10 taxa per sample type (by mean relative abundance) are also annotated, along with their prevalence among the samples for each sample type.

To explore the variation in the microbiome composition between sample types, we compared the relative abundance and presence/absence of microbial lineages across all samples (Fig. 4 to 7; Table S4). Comparison of phyla with a mean relative abundance ≥1% in at least one sample type revealed that *Actinobacteriota* was significantly lower in SCC compared to those in NS and SCC_PL (*P ≤* 0.01) (Fig. 4 to 6; Table S4). The SCC_PL and SCC samples had a significantly lower abundance of *Proteobacteria* than NS (*P ≤* 0.01), and SCC contained a significantly higher abundance of *Firmicutes* than NS (*P ≤* 0.01). The other phylum *Bacteroidota* was not significantly different between SCC, SCC_PL, and NS.

When considering the microbial profiles of all sample types, regardless of pet species, there are several significant differences between NS, SCC_PL, and SCC samples at the genus level (Table S4). Normal skin had significantly higher levels of *Psychrobacter* and *Conchiformibius* than SCC_PL and SCC samples (*P ≤* 0.001–0.05). SCC_PL had significantly higher *Cutibacterium*, *Corynebacterium,* and the fungus *Malassezia* than SCC (*P ≤* 0.001–0.01). When analyzing the significant differences between SCC_PL and SCC skin, it appears that SCC_PL exhibits more similarities to NS than SCC skin due to the presence of these genera.

*Staphylococcus* was detected in all sample types but elevated in SCC_PL and SCC samples; 16% (4/25) of SCC_PL and 50% (6/12) SCC samples had >50% relative abundance of this genus (Fig. 4 and 7; Fig. S7). At the species level, *S. aureus*, *S. felis*, and *S. pseudintermedius* were in higher relative abundance in SCC (mean abundance of 9.3%, 26.8%, and 6.9%, respectively) and SCC_PL (5.2%, 6.7%, and 7.2%, respectively) samples compared to those in NS (0.01%, 2.5%, and 1.4%, respectively); however these differences were not significant (Fig. 6).

### The core microbiota of normal pet skin is composed of commensal genera, while SCC and SCC_PL include pathogenic genera

The analysis of the core microbiota across all sample types revealed that each type had genera unique to itself as well as genera in common with the other sample types (Fig. 7; Table S5). The NS samples had 46 unique genera, whereas the SCC_PL and SCC samples had 53 and three, respectively. The SCC_PL and NS samples had substantial overlap, sharing 44 genera. NS and SCC_PL samples had the highest number of unique genera; however, this may be due to the higher number and increased hair density of samples compared to those of the other sample types. SCC and SCC_PL skin shared eight genera, including several potentially pathogenic genera such as *Filifactor* and *Peptostreptococcus* (Table S5). Unexpectedly, the bacterial genus *Candidatus Methylomirabilis* was detected at low abundance (approximately 0.004%–1.8%) on NS and SCC_PL samples from several pets. This bacterium has been found in a range of environmental samples and can couple anaerobic methane oxidation with nitrite reduction in anoxic habitats (39–42). To the best of our knowledge, this is the first time this organism has been identified on the skin or in association with an animal host. It is not known if this unusual bacterium colonized the animals or is the result of environmental exposure. All sample types contained the common skin commensal *Cutibacterium*, which has been previously found in pets as well as in humans (43, 44).

**Fig 8:**
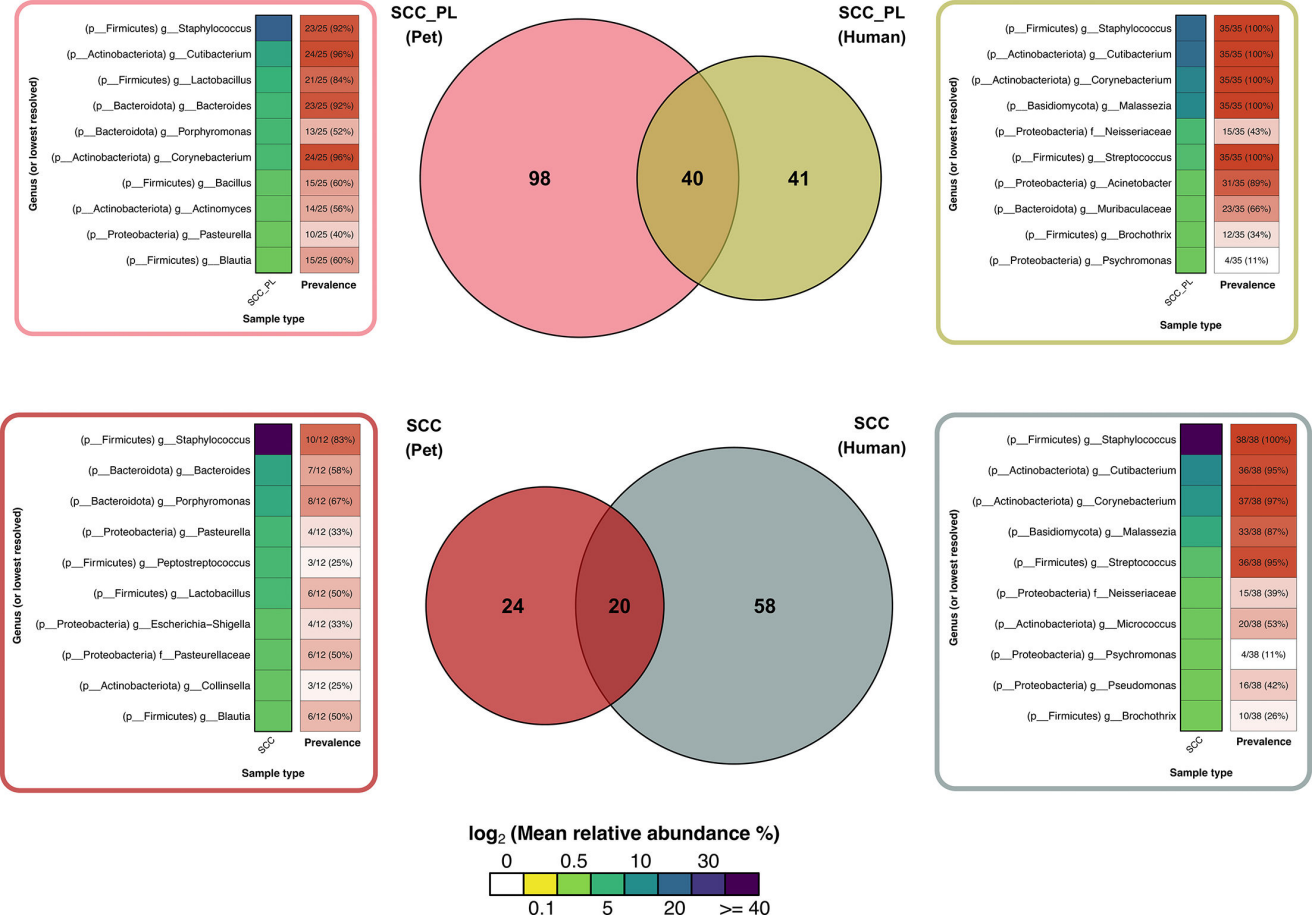
Venn diagrams showing the number of shared microbes across pet and human SCC and SCC_PL sample types. The core microbiome was calculated across pet and human sample types and defined as those genera that appeared across types (pet and human SCC, or pet and human SCC_PL), and per type and host (pet or human), were present in at least two samples, and had a relative abundance of 1% in at least one sample. The mean relative abundances of the top 10 taxa per sample type (by mean relative abundance) are also annotated, along with their prevalence among the samples for each sample type.

To assess the potential of using companion animals as a translatable model for human microbiome-related SCC progression, the microbiomes of the SCC and SCC_PL samples from this study were compared to published immunocompetent human SCC and SCC_PL microbiome data (11, 12). A core microbiome analysis revealed that SCC and SCC_PL pet and human sample types shared 20 and 40 genera, respectively (Fig. 8; Table S6). *Staphylococcus* was the most prevalent genera for all sample types, with 83% (10/12) of the SCC and 92% (23/25) of the SCC_PL pet samples and 100% (38/38) SCC and 100% (35/35) of the SCC_PL human samples with *Staphylococcus* at a high mean relative abundance (19%–48%) (Fig. 8). *Corynebacterium* and *Cutibacterium* were also highly prevalent, both of which were in 96% (24/25) and 100% (35/35) of the SCC_PL samples from pets and humans, respectively.

## DISCUSSION

While numerous studies have sought to define a healthy microbiome for various cutaneous regions in cats and dogs, their results have often varied and typically excluded the nasal planum (45–47). The microbiome of cutaneous squamous cell carcinoma (SCC) in cats and dogs has also remained uncharacterized. To address this knowledge gap, we characterized the skin microbiomes of healthy pets and those with cutaneous SCC. Historically, researchers have used culture based-techniques to characterize the skin microbiome, but these methods were limited in accurately identifying the growth of fastidious organisms (48). With the advent of culture-independent techniques, such as SSU rRNA sequencing, we now have the capacity to detect a broader range of organisms. Therefore, this study used SSU rRNA sequencing to gain a comprehensive view of the microbiome associated with SCC in cats and dogs.

We found that normal skin samples had high microbial diversity, whereas the samples from SCC lesions had lower diversity with an over-representation of *Staphylococcus* species (Fig. 4). NS samples also contained a variety of genera, such as *Moraxella*, *Cutibacterium*, and *Malassezia,* that are commensal to human skin. *Malassezia* is a yeast commonly found on healthy cats and dogs in low-to-moderate abundance (49–51). High levels of *Malassezia* can be indicative of dermatological diseases in canines and felines as it is an opportunistic pathogen (52). Another human skin commensal, *Corynebacterium*, was found to be significantly more abundant on SCC_PL skin compared to SCC skin in the present study (*P ≤* 0.001). This genus is present on normal human skin; however, it has been associated with canine atopic dermatitis when in high abundance (47). While not significant, the alpha diversity was reduced in the SCC_PL samples compared to NS (Fig. 2), suggesting that SCC_PL skin could be in a transitional state. The significant reduction in these commensal species on SCC lesions highlights microbial dysbiosis occurring in affected pets.

Staphylococcal species, notably *S. felis* and *S. pseudintermedius*, dominated a number of the SCC samples (Fig. 6; Fig. S7). These opportunistic pathogens are linked to various veterinary and human diseases. Older et al. hypothesized that staphylococcal species may contribute to feline allergic dermatitis (53). In particular, *S. felis*, found at higher abundance in allergic lesional skin compared to that in normal cat skin, may play a similar role to that of *S. aureus* in human atopic dermatitis. Ma and colleagues found that of four swabbed unspecified skin lesions in cats, *S. felis* contributed to 75% of the total staphylococcal carriage, with the remaining 25% being attributed to *S. pseudintermedius* (54). The present study has presented results consistent with these findings, as both *S. felis* and *S. aureus* are found in high abundance on some SCC lesions and perilesional skin in cats, whereas SCC lesions and perilesional skin in dogs typically had a high abundance of *S. pseudintermedius* and *S. aureus*.

*Janibacter* and *Herpetosiphon*, two bacterial genera, were identified on nine samples across seven individuals (six dogs and one cat), with over 1% relative abundance on one canine SCC lesion (Table S5; Fig. S8). Neither of these bacteria have been widely reported in humans nor have there been any known reports of their presence on companion animals. There have been several case reports published of *Janibacter* species causing bacteraemia in humans, with the first reported case in 2005 (55–59). This genus comprises opportunistic pathogens that were first isolated from the upper layers of the atmosphere but also reside in soil (60). A systematic review and meta-analysis conducted by Peter et al. on the oral SCC-associated microbiome found that *Janibacter* was more abundant in the control population than in the oral SCC population (61). *Herpetosiphon* is a genus comprised of at least four species known for their filamentous gliding motility and ability to prey on other microbes including several staphylococcal species (62, 63). It is unknown whether the filamentous nature of *Herpetosiphon* enables it to move across the SCC surface; however, it could reside within an SCC biofilm, along with several other biofilm-forming bacteria detected within the lesions of *Staphylococcus*, *Janibacter*, and *Filifactor* (64–67). This genus also produces secondary metabolites that contain antimicrobial compounds that could be further examined for utilization as a topical microbiome-based therapy (62, 68). No correlation was observed between *Janibacter* and *Herpetosiphon* and the staphylococcal species in the samples from the present study (Fig. S8).

*Peptostreptococcus* and *Filifactor* were two of the genera shared between SCC_PL and SCC samples (Table S5). Peter et al. found that these two genera were abundant in oral SCC patients compared to the control population (61). *Filifactor* species, *F. villosus* and *F. alocis,* are pathogenic biofilm-generating bacteria that are positively correlated with canine and human periodontal disease (64, 69, 70). Similarly, *Peptostreptococcus* has been found in the oral cavity of cats and dogs with supragingival plaque (71, 72) and in the ears of canines with otitis externa (43). However, the pathogenic status of this bacterial genus has not been established, so here, we report it only as potentially pathogenic. *Peptostreptococcus* was also found as a commonality of pet and human SCC in the core microbiome analysis (Table S6). These bacteria may be enriched in pets with SCC due to behavioral attributes such as regular licking of the SCC wound. The pets affected by SCC had a median age of 12 years old and were in the late stage of disease progression and had “open” wounds. Licking of wounds is a behavioral response aimed at cleaning the wound of foreign materials, tissue debris, and bacterial contaminants (73). Elderly pets are also highly susceptible to dental disease, with 60% of dogs having periodontal disease and 85.3% having dental alterations, which could explain the presence of *Peptostreptococcus* and *Filifactor* observed on SCC lesions (74).

Similarities between the human and companion animal skin microbiome have been hypothesized to occur due to co-habitation and domestication (75, 76). Wetzels et al. found that the similarity of the skin microbiome between canines and humans was dependent on the level of contact between the species, indicating that microbes living on the skin of both hosts may be transferrable (75). This finding strengthens the hypothesis that companion animals could be a suitable model for understanding the role of microbiota in human skin cancer; however, no studies that we are aware of have addressed the relative effects of inter-species skin microbiome transfer vs inherent properties of SCCs that may promote colonization with similar taxa such as *Staphylococcus*.

However, there are significant considerations to make when contemplating the use of pets for experimental purposes (77). Historically, the use of animals in research, particularly companion animals, has not been widely accepted by the public; indeed, there is increasing pressure for researchers to reduce animal usage (78). It is likely that pets could be used in place of a human observational or interventional study once initial *in vitro* experimentation and safety data have been generated. *In vitro* data are essential for initial testing to establish pathway mechanisms or biological effects of a new intervention on the intended target, but current accessible *in vitro* models are limited in terms of translatability into a living organism (79). Utilizing pets in this way would mimic a natural disease progression in an environment similar to humans, with indoor and outdoor exposure that would not occur in a laboratory animal with an induced disease. Thus, pets with diseases, such as cutaneous SCC, would have access to new trial interventions for disease treatment that may improve overall outcomes for the animal.

We acknowledge that these observations are limited by the small sample size, with *n* = 13 SCC lesions swabbed and *n* = 12 analyzed after quality control; therefore, further studies with a larger sample size are required to substantiate and extend our observations.

### Potential relevance to human SCC

There is significant debate within the scientific community as to whether laboratory mice are an appropriate model organism for the study of *S. aureus* associated with human disease and infection (17). Typically, murine models have been used to model infection in humans; however, translating *S. aureus*-related findings from mice to human medicine has proven challenging, despite its reputation as a multi-host pathogen (16). *S. aureus* exhibits substantial host tropism, with a large variety of clonal complexes that have delineated, resulting in a diverse family tree of *S. aureus* strains. Mice and humans share some physiological similarities; however, as discussed extensively in a recent review, the constitution and function of their immune systems and microbiomes exhibit key differences, and establishing *S. aureus* in mice requires a high infectious dose and intensive labor (16). Additionally, Mrochen et al. noted that there are host species-specific staphylococcal toxins, adhesins, nutrient acquisition systems, and mechanisms to evade the immune system (16). Although there is increasing evidence in humans to suggest that microbial dysbiosis may play a role in cutaneous SCC disease progression, there are no published studies of this in companion animals to our knowledge, and there are limited options to investigate microbial dysbiosis relevant to humans using animal models.

A study by Ross et al. compared the microbiome of humans with 38 other mammalian species, including cats and dogs, and found that the human microbiome most closely overlaps with domestic cats and dogs compared to the other mammals investigated (80). Recent studies by Krueger and colleagues found that secreted products from AK and SCC-associated *S. aureus* caused overexpression of inflammatory mediators linked to carcinogenesis and induce oxidative stress-linked DNA damage in human keratinocytes *in vitro* (14, 15). Future investigations into the effect of the various staphylococcal species isolated from pet SCC on *in vitro* tissue cultures should be conducted to determine whether toxins contributing to carcinogenesis are being produced by pet-specific *Staphylococcus* strains. As companion animals also exhibit microbial dysbiosis on lesional skin, with *Staphylococcus* species being enriched on SCC lesions, this presents a new opportunity to utilize companion animals for studying the malignant progression of AK lesions and testing novel therapies for both human and veterinary medicine.

### Conclusion

This study is the first to investigate the microbiome of cutaneous SCC in cats and dogs using culture-independent molecular profiling. Compared to normal skin, SCC lesions have significantly less microbial diversity, and an increased abundance of *Staphylococcus* species likely contributing to microbial dysbiosis. In humans with AK and cutaneous SCC, microbial dysbiosis with an overabundance of *S. aureus* has been demonstrated. While further work is required with a larger cohort of cats and dogs, companion animals may be a suitable model to test new therapies applicable to humans due to the similarity in microbial dysbiosis.

## Data Availability

ASV counts and associated metadata for all pet samples have been provided in the supplementary material (Table S1). Amplicon sequencing data have been deposited in the NCBI Sequence Read Archive (https://www.ncbi.nlm.nih.gov/sra) under the BioProject PRJNA949396.

## SUPPLEMENTAL MATERIAL

Supplemental material msphere.00555-23-s0001.pdf Supplemental text, figures, and table legends.

Table S1 msphere.00555-23-s0002.xlsx ASV counts and associated metadata for all pet samples.

Table S2 msphere.00555-23-s0003.xlsx Percent contributions to the variance for PCs 1 and 2 and ordination axes by genera present in all and nasal planum pet samples.

Table S3 msphere.00555-23-s0004.xlsx PERMANOVA and PERMDISP results for all and nasal planum pet samples.

Table S4 msphere.00555-23-s0005.xlsx Differentially abundant ASVs.

Table S5 msphere.00555-23-s0006.xlsx Genera shared between, and unique to, NS, SCC_PL, and SCC sample types, across all pet samples.

Table S6 msphere.00555-23-s0007.xlsx Genera shared, and unique to, SCC_PL and SCC sample types, across pet and human samples.

ASM does not own the copyrights to Supplemental Material that may be linked to, or accessed through, an article. The authors have granted ASM a non-exclusive, world-wide license to publish the Supplemental Material files. Please contact the corresponding author directly for reuse.
