# Supplementary material for: Characterization of the skin microbiome in normal and cutaneous squamous cell carcinoma affected cats and dogs
Source: mSphere. 2024 Mar 26;9(4):e00555-23. doi: 10.1128/msphere.00555-23 (PMC11036808; doi:10.1128/msphere.00555-23)
Supplement: Supplemental material — Supplemental text, figures, and table legends. [file msphere.00555-23-s0001.pdf]

Supplementary material

Supplementary Text S1

Unprocessed amplicon sequence variant (ASV) count data and corresponding metadata for squamous cell carcinoma (SCC, n = 38) and matching perilesional control (SCC_PL, n = 35) swab samples from a cohort of immunocompentent human subjects (1) were generated in the study of A. Krueger et al. (2). Consistent with the pet samples from the present study, the SSU rRNA genes for these samples were also PCR amplified with universally conserved primers (926F/1392R). Human sample ASV data were processed similarly to the pet samples: ASVs that were not bacterial, fungal or archaeal in origin, classified at below the phylum level, or that were classified as chloroplast or mitochondria, were discarded. The decontam package (ver. 1.20.0; method = prevalence, threshold = 0.6) (3) was used to identify and remove likely contaminant ASVs that were more prevalent in negative control swabs (n = 33). Low-depth samples with less than 1,000 reads were then removed.

References

1. Wood DLA, Lachner N, Tan J-M, Tang S, Angel N, Laino A, Linedale R, Lê Cao K-A, Morrison M, Frazer IH, Soyer HP, Hugenholtz P. 2018. A Natural History of Actinic Keratosis and Cutaneous Squamous Cell Carcinoma Microbiomes. mBio 9:1033.
2. Krueger A, Zaugg J, Lachner N, Bialasiewicz S, Lin LL, Gabizon S, Sobarun P, Morrison M, Soyer HP, Hugenholtz P, Frazer IH. 2022. Changes in the skin microbiome associated with squamous cell carcinoma in transplant recipients. ISME Communications 2:13.
3. Davis NM, Proctor DM, Holmes SP, Relman DA, Callahan BJ. 2018. Simple statistical identification and removal of contaminant sequences in marker-gene and metagenomics data. Microbiome 6:226.

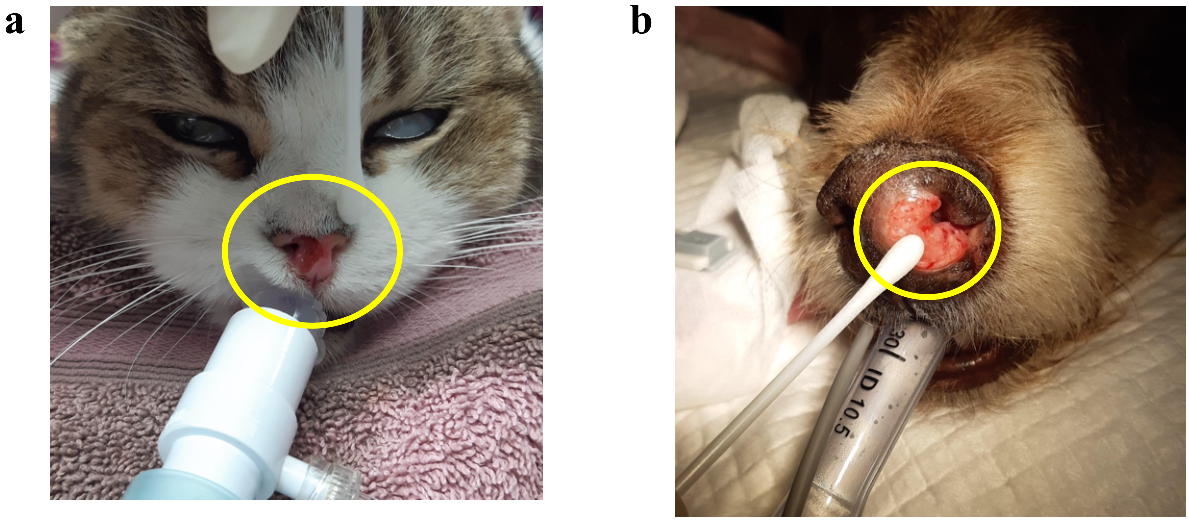

Supplementary Figure S1: (**a**) Nasal planum of cat with SCC lesion (Hazel Rodriguez, female, de-sexed, 13 years old) and **(b)** Nasal planum of dog with SCC lesion (Judd Bower, male, desexed, 9 years old).

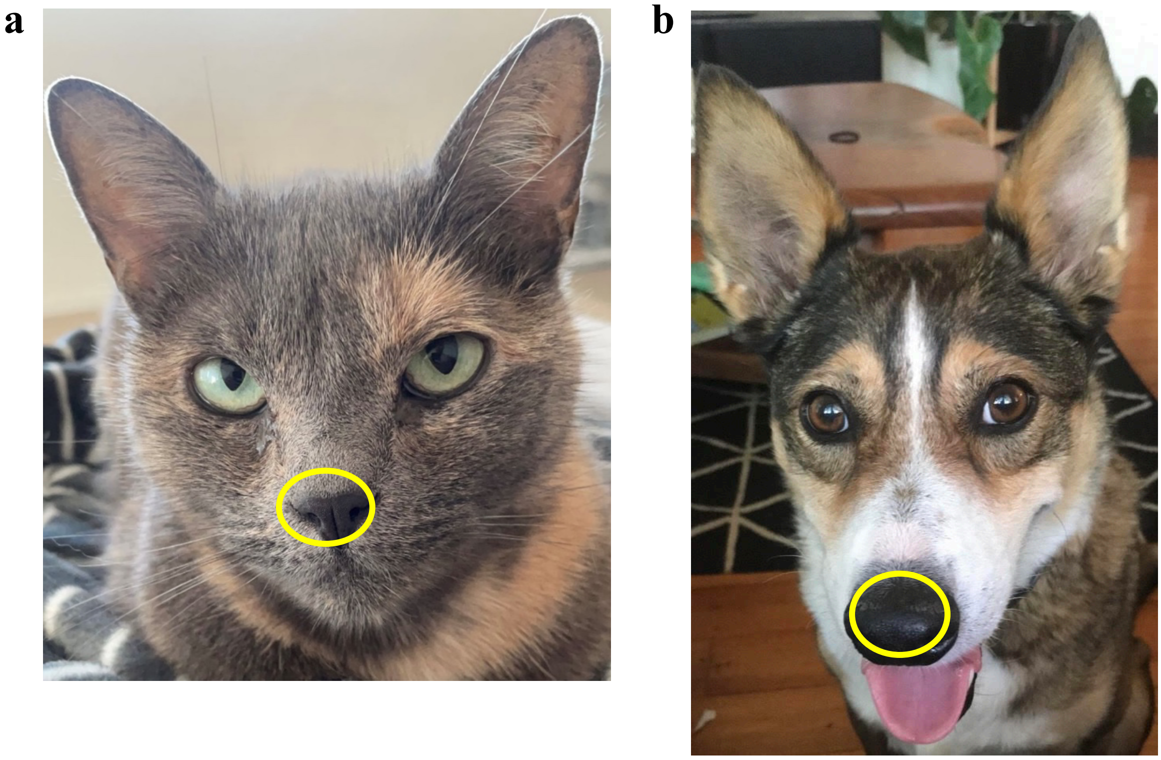

Supplementary Figure S2: (a) Nasal planum of a healthy cat (Katzi Krueger, female, de-sexed, 4 years old) and **(b)** dog (Morris Bromfield, male, de-sexed, 10 years old).

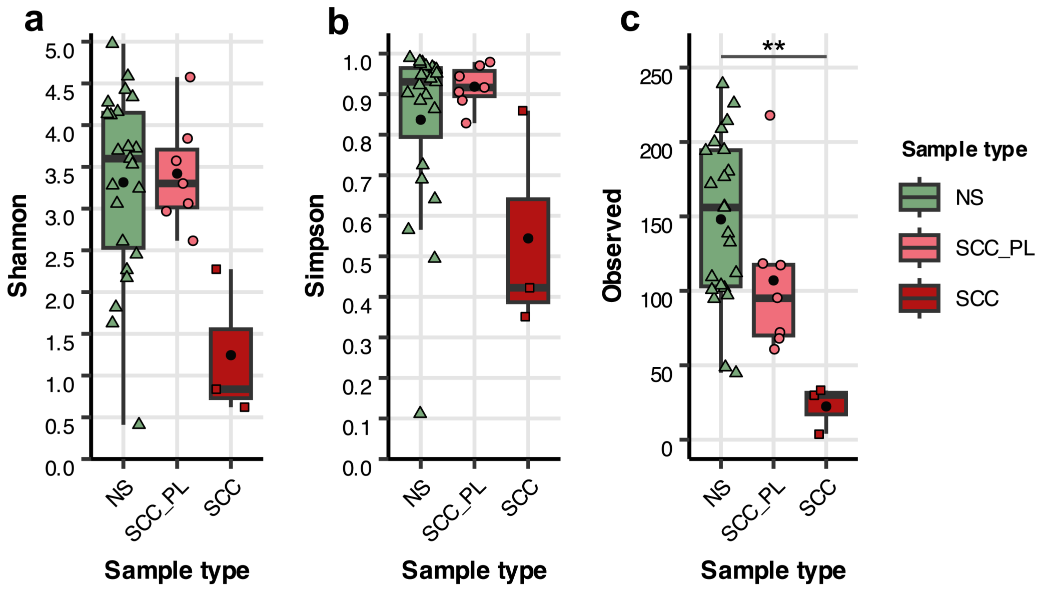

Supplementary Figure S3: Alpha diversity in nasal planum normal skin (NS), squamous cell carcinoma (SCC) and matching perilesional control (SCC_PL) swabs from the pet cohort. Tukey style box plots showing genus diversity across sample types, with diversity represented by three separate measures: **(a)** Shannon (diversity), **(b)** Simpson (evenness) and **(c)** number of observed taxa. Bars indicate median +/- 1.5 × interquartile range and the mean diversity for each sample type is indicated by the black dot. Significant differences between sample types are indicated by * = P ≤ 0.05, ** = P ≤ 0.01 and *** = P ≤ 0.001 as calculated via Dunn's multiple comparisons test.

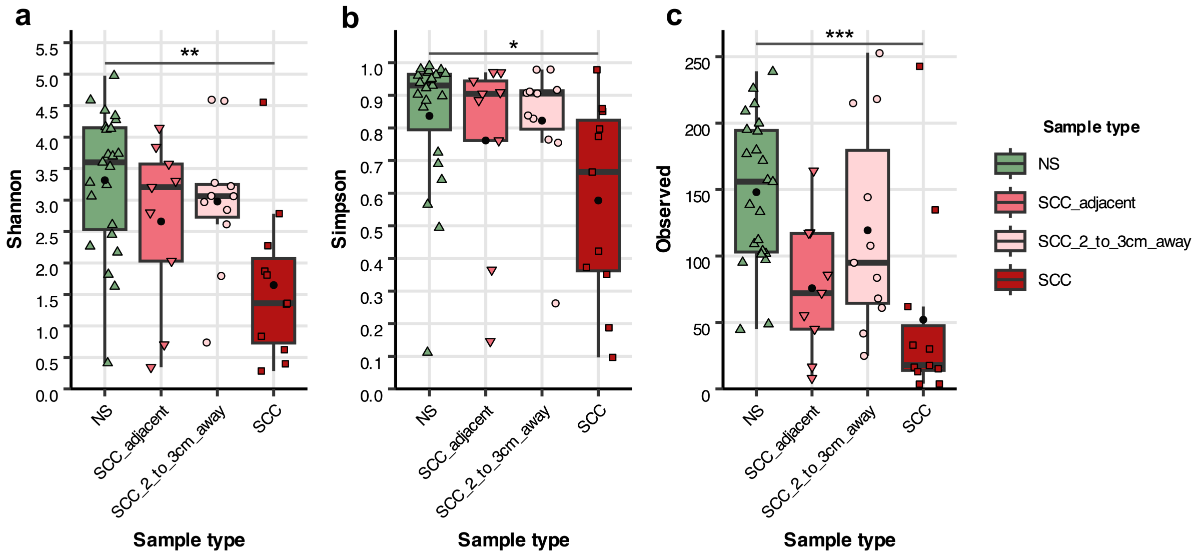

Supplementary Figure S4: Alpha diversity in normal skin (NS), squamous cell carcinoma (SCC), and matching perilesional control swabs (adjacent and 2–3cm away) from the pet cohort. Tukey style box plots showing genus diversity across sample types, with diversity represented by three separate measures: **(a)** Shannon (diversity), **(b)** Simpson (evenness) and **(c)** number of observed taxa. Bars indicate median +/- 1.5 × interquartile range and the mean diversity for each sample type is indicated by the black dot. Significant differences between sample types are indicated by * = P ≤ 0.05, ** = P ≤ 0.01 and *** = P ≤ 0.001 as calculated via Dunn's multiple comparisons test.

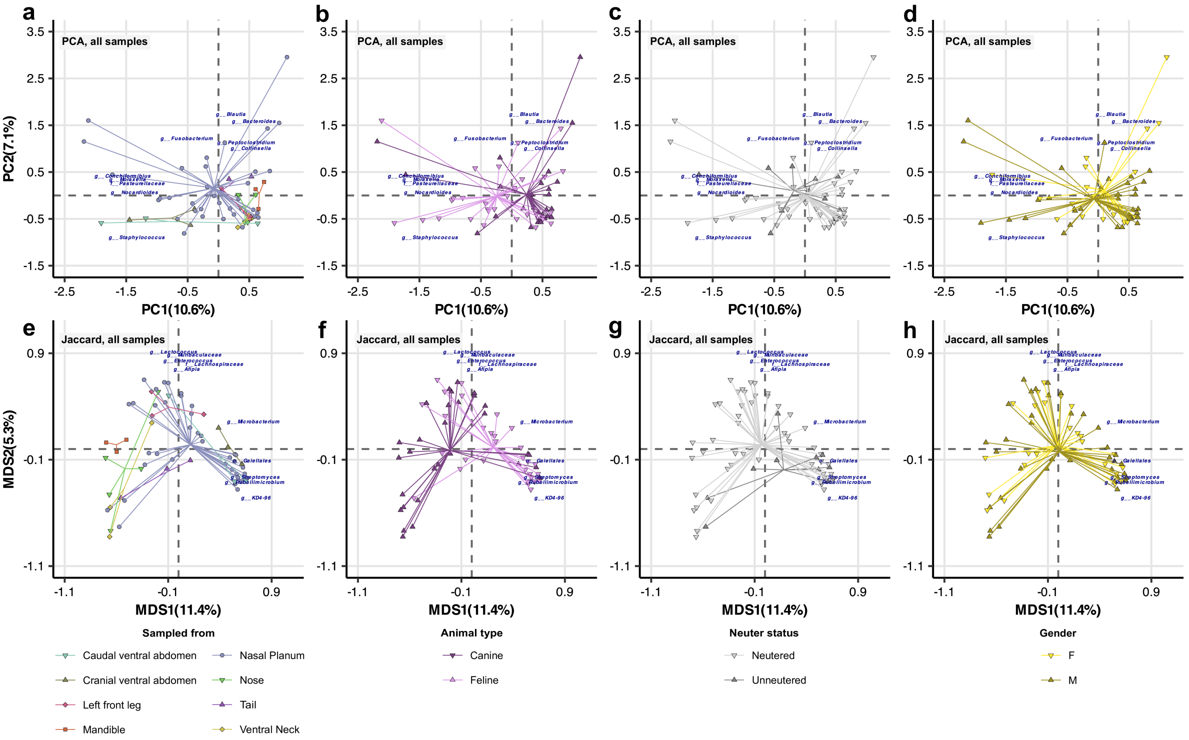

Supplementary Figure S5: Principal-component analysis (PCA) of the Euclidean distances for robust centred log-ratio (rclr) values, and ordination analysis of Jaccard dissimilarities, visualising the variation in microbial community composition across skin swab samples for the pet cohort. PCA and Jaccard ordinations based on all samples, annotated by **(a,e)** sampled location, **(b,f)** animal type, **(c,g)** neuter status and **(d,h)** gender.

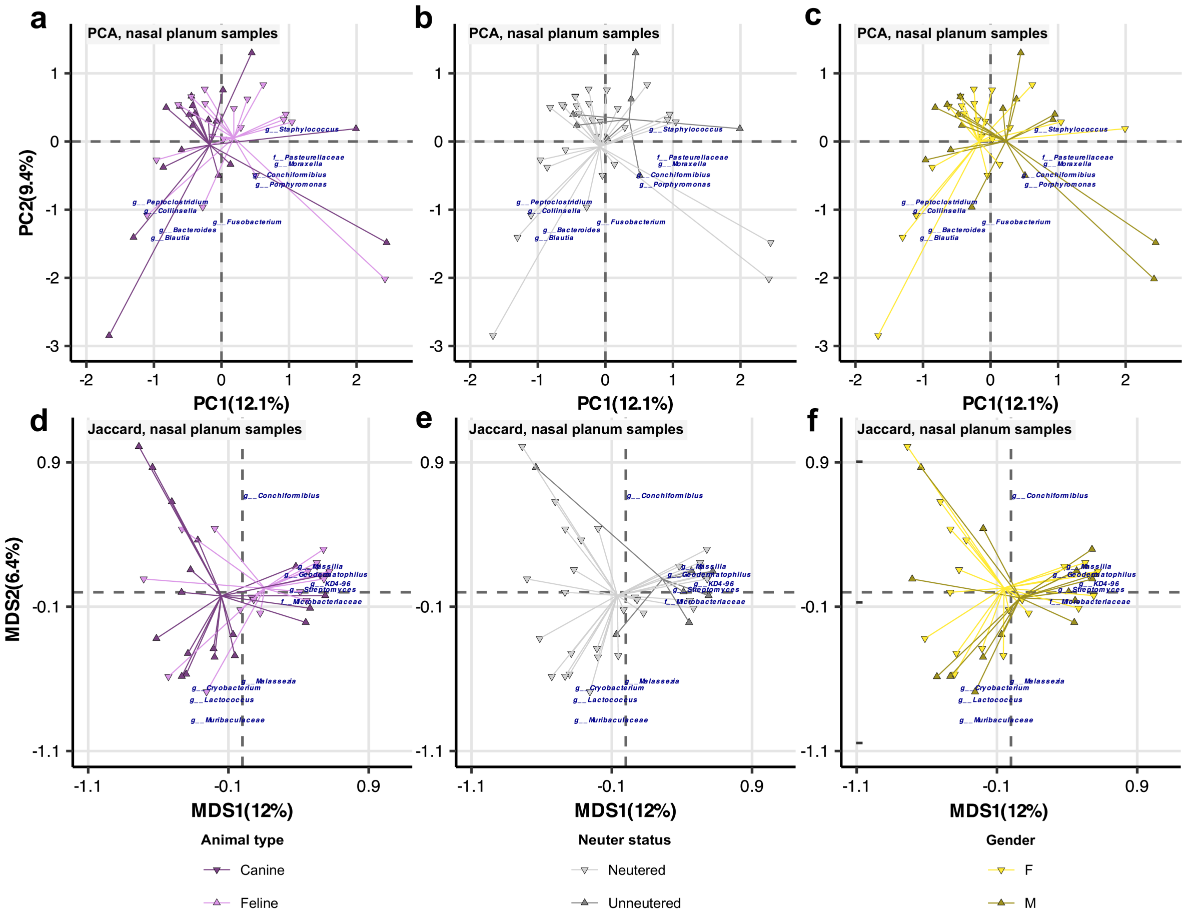

Supplementary Figure S6: Principal-component analysis (PCA) of the Euclidean distances for robust centred log-ratio (rclr) values, and ordination analysis of Jaccard dissimilarities, visualising the variation in microbial community composition across skin nasal planum swab samples for the pet cohort. PCA and Jaccard ordinations based on nasal planum samples, annotated by **(a,d)** animal type, **(b,e)** neuter status and **(c,f)** gender.

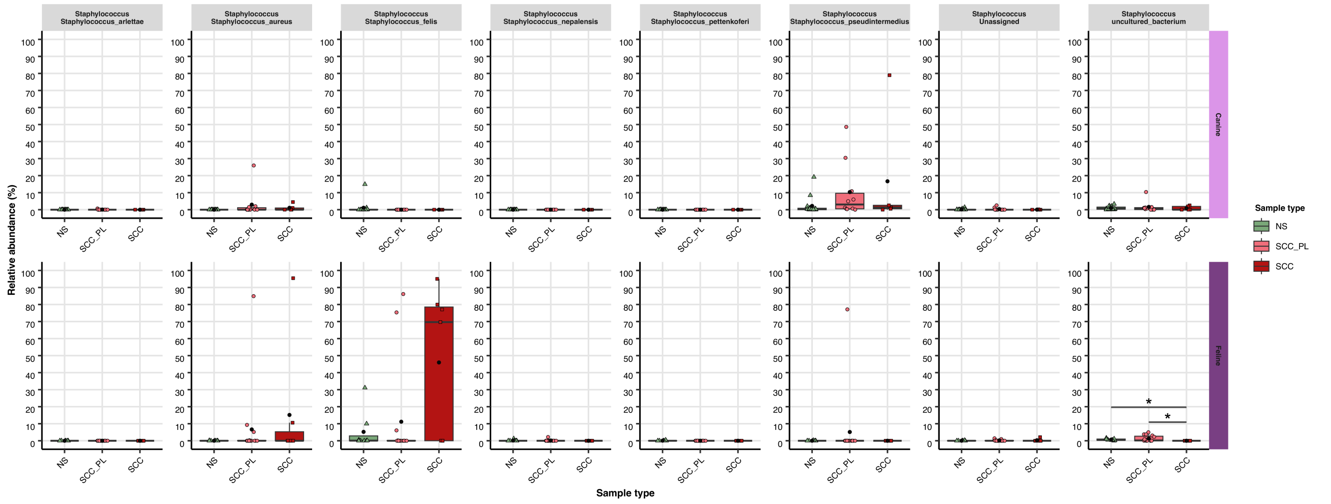

Supplementary Figure S7: Relative abundance of staphylococcal species across sample types, for both animal types (canine and feline). Tukey style box plots showing the relative abundances (normalised SSU rRNA read counts) of the staphylococcal species in swab samples, per sample and animal type. Bars indicate the median +/- 1.5 × interquartile range and the mean relative abundance for each sample type is indicated by the black dot. Significant differences between sample types are indicated by * = P ≤ 0.05, ** = P ≤ 0.01 and *** = P ≤ 0.001 as calculated via Dunn's multiple comparisons test.

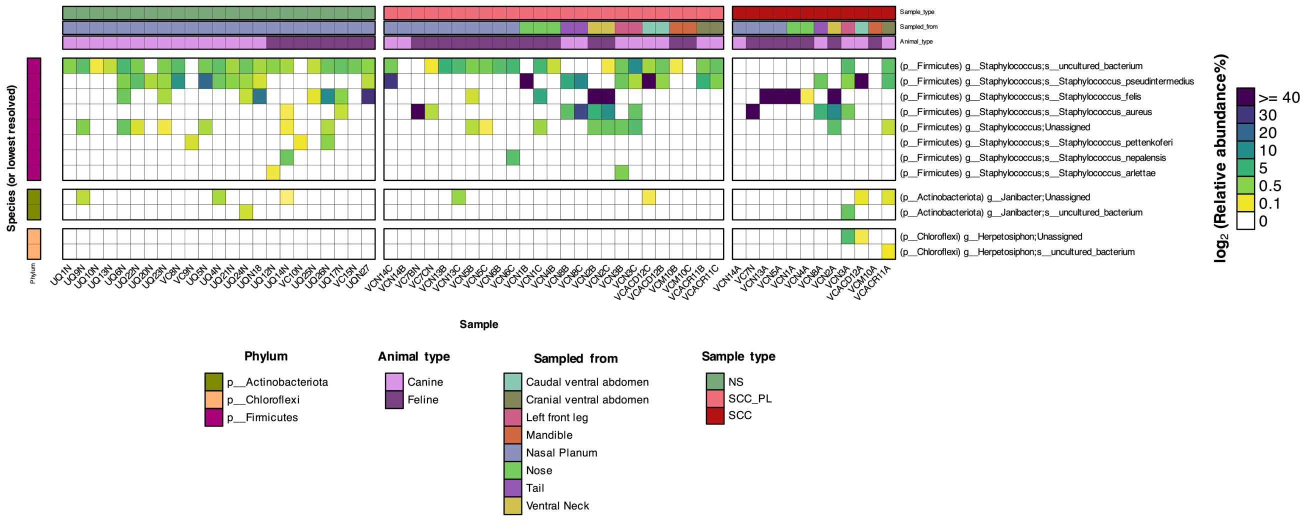

Supplementary Figure S8: Heatmap of the relative abundances of *Janibacter*, *Herpetosiphon* and staphylococcal species across the pet cohort.

Supplementary Table legends

Supplementary Table S1: Amplicon Sequence Variant (ASV) counts and associated metadata for all pet samples.

Supplementary Table S2: Percent contributions to the variance for principal components (PCs) 1 and 2 and ordination axes (from analysis of Jaccard dissimilarities) by genera present in all and nasal planum pet samples.

Supplementary Table S3: Permutational multivariate analysis of variance (PERMANOVA) and permutation tests for homogeneity of multivariate dispersions (PERMDISP) results for all and nasal planum pet samples.

Supplementary Table S4: Differentially abundant ASVs, species, genera and phyla identified by ALDEx2 and LinDA, and through the comparison of relative abundance distributions with Kruskal-Wallis tests followed by Benjamini & Hochberg corrected Dunn's multiple comparisons tests.

Supplementary Table S5: The genera shared between, and unique to, NS, SCC_PL and SCC sample types, across all pet samples.

Supplementary Table S6: The genera shared, and unique to, SCC_PL and SCC sample types, across pet and human samples.
